# Prevalence and correlates of truancy among adolescents in Swaziland: findings from the Global School-Based Health Survey

**DOI:** 10.1186/1753-2000-1-15

**Published:** 2007-11-23

**Authors:** Seter Siziya, Adamson S Muula, Emmanuel Rudatsikira

**Affiliations:** 1 Department of Community Medicine, School of Medicine, University of Zambia, Lusaka, Zambia; 2 Department of Community Health, College of Medicine, University of Malawi, Blantyre, Malawi; 3 Departments of Epidemiology and Biostatistics and Global Health, School of Public Health, Loma Linda University, Loma Linda, California, USA

## Abstract

**Background:**

Educational attainment is an important determinant of diverse health outcomes. Truancy among adolescents jeopardizes chances of achieving their educational goals. Truant behaviors are also associated with various psychosocial problems. There is however limited data on the prevalence and factors associated with truancy among adolescents in Africa.

**Methods:**

We used data from the Swaziland Global School-Based Health Survey (GSHS) conducted in 2003 to estimate the prevalence of self-reported truancy within the last 30 days among adolescents. We also assessed the association between self-reported truancy and a selected list of independent variables using logistic regression analysis.

**Results:**

A total of 7341 students participated in the study. In analysis of available data, 2526 (36.2%) and 4470 (63.8%) were males and females respectively. The overall prevalence of truancy within the last 30 days preceding the study was 21.6%. Prevalence of truancy was 27.4% (605) and 17.9% (723) in males and females respectively. In multivariate logistic regression analysis, being a male, having been bullied, lower school grades, and alcohol use were positively associated with truancy. Adolescents who perceived themselves as having parental support were less likely to have reported being truant.

**Conclusion:**

Truancy among adolescents in Swaziland should be regarded as an important social problem as it is relatively prevalent. The design and implementation of intervention programs aimed to reduce truant behaviours should incorporate our knowledge of the factors identified as associated with bullying.

## Background

Educational attainment is a crucial predictor of several health-related lifestyles and premature mortality. However truant behaviours have potential to curtail possibilities of meaningful academic achievement. Truancy is a predictor of multiple health risk behaviours among adolescents. Truant adolescents have been reported to engage in risky sexual practices, illicit drug use, alcohol drinking and cigarette smoking [1-4]. Henry [5] has suggested that the unsupervised time that adolescents have when they are truant allows them to initiate and maintain unhealthy behaviours.

Truancy in childhood may be associated with adverse social and health outcome later in life. Studies have reported that adults who were truant as adolescents were more likely to experience marital or job instability and psychosocial maladjustment when compared to their counterparts who were not truant as adolescents [6-8].

A 1990 study by Obondo and Dhadphale reported that about 10% of school non-attendance by children in Kenya was due to truancy [9]. Olley studied 169 street youths in Ibadan, Nigeria [10] and about 47% of these had a history of truancy. These studies suggest an association between truancy and being on the streets as well as that truancy is an important contributor of non-attendance at school.

Other factors that have been reported as associations with truancy are level of parental education, amount of adolescents' unsupervised time, poor school grades and illicit drug use [5]. In order to highlight the significance of truancy in the social discourse in developing countries, there is need to estimate its prevalence and associated factors. There is however limited information about the prevalence of truancy among adolescents in Africa. We believe knowledge about this estimate and associated factors will inform public health and educational policies. We therefore conducted a secondary analysis of the Swaziland Global School-Based Health Survey (GSHS) in order to obtain estimates of prevalence and correlates of truancy among adolescents.

## Methods

Our study involved secondary analysis of existing data from the Swaziland Global School-Based Health Survey (GSHS) conducted in 2003. The GSHS was developed by the World Health Organization (WHO) in collaboration with United Nations' UNICEF, UNESCO, and UNAIDS with technical assistance from the Centers for Disease Control and Prevention (CDC). The GSHS aims to provide data on health and social behaviours among school-going adolescents.

The GSHS used a two-stage probability sampling technique. In the first stage, primary sampling units were schools which were selected with a probability proportional to their enrolment size. In the second step, a systematic sample of classes in the selected school was obtained. All students in the selected classes were eligible to participate. A self-completed questionnaire was used. A total of 7647 students were eligible to participate; however only 7341 actually did (96%). The school response rate was 97%. Truancy was defined as missing classes without permission within the last 30 days preceding the survey. Students were asked: "During the past 30 days, on how many days did you miss classes or school without permission?" A full presentation of the questions that were considered is presented in Table 1.

**Table 1:** Variables considered in the analysis

**Outcome variable**
During the past 30 days, on how many days did you miss classes or school without permission? (Yes, No)

**Explanatory variables**
1. Age (11–13, 14, 15+ years)
2. Sex (Male, Female)
3. Grade (Grades 6 & 7 Form1, Forms 2–4)
***In the following factors, the "Never or Zero" category was recoded as "No" and the rest of the categories as "Yes"***
4. During the past 30 days, how often did you go hungry because there was not enough food in your home? (Yes, No)
5. During the past 30 days, on how many days were you bullied? (Yes, No)
6. During the past 30 days, on how many days did you have at least one drink
containing alcohol? (Yes, No)
7. During the past 30 days, how often were most of the students in your school kind and helpful? (Yes, No)
8. During the past 30 days, how often did your parents or guardians check to see if your homework was done? (Yes, No)
9. During the past 30 days, how often did your parents or guardians understand your problems and worries? (Yes, No)
10. During the past 30 days, how often did your parents or guardians really know what you were doing with your free time? (Yes, No)

### Data analysis

Data analysis was performed using SPSS software, version 14.0 (Chicago, IL, United States). A weighting factor was used in the analysis to reflect the likelihood of selection of each student into the sample and to reduce bias by compensating for differing patterns of non response. The weight used for estimation of prevalence estimates is given by the following formula:

W = W1 * W2 * f1 * f2 * f3 * f4

where W1 = the inverse of the probability of selecting the school

W2 = the inverse of the probability of selecting the classroom within the school

f1 = a school-level non response adjustment factor calculated by school size category (small, medium, large)

f2= a class-level non response adjustment factor calculated for each school

f3 = a student-level non response adjustment factor calculated by class

f4 = a post stratification adjustment factor calculated by grade

We obtained frequencies and weighted proportions to estimate the prevalence of truancy and other socio-demographic characteristics. We also conducted backward logistic regression analysis to estimate the association between relevant predictor variables and truancy. In the calculation of odd ratios for any one particular predictor variables, the other predictor variables were controlled for.

### Study Setting

The Kingdom of Swaziland is a southern African country that is almost totally surrounded by the Republic of South Africa except on its eastern side where it shares borders with Mozambique. The country has an estimated population of about 1.3 million.

Primary education runs forseven years with an entry age at 6 years. The seventh year is externally examined by the Examinations Council of Swaziland (ECOS) and these examinations serve as a selection tool for students to proceed to junior secondary education. Junior secondary education takes three years and culminates in the Junior certificate qualifying examinations administered by a national examinations board. The final phase of secondary school is 2 years and students sit for Cambridge O level examinations (UK) [11]. School fees are charged and lack of it may result in drop out. Although the government aims towards universal education, this is not compulsory and is hampered by limited resources. The infant mortality is estimated at 78 deaths per 1,000 live births. Unemployment rate was estimated at 22.8% for 2003 [12].

## Results

### Characteristics of the study participants

Altogether 7341 students participated in the survey. Most of the students were females (63.8%), were of age 15 years or more (42.5%), and were in their 6^th ^to 8^th ^year of schooling (65.3%). Overall, 39.6% of the adolescents were bullied on one or more days in the last 30 days. About a quarter (25.2%) of the adolescents felt that most students were never kind and helpful, and 16.6% drank alcohol. The majority (31.3%) of parents never supervised their children's homework. Further description of the sample is depicted in Table 2. The overall prevalence of truancy was 21.6%.

**Table 2:** Characteristics of study participants in the Swaziland Global Health Survey, 2003

**Factor**	**Total**	**Males**	**Females**
	n* (%)**	n* (%)**	n* (%)**
**Age**			
< 14	1465 (20.6)	472 (18.8)	949 (21.7)
14	2586 (36.9)	871 (35.5)	1635 (37.6)
15+	3049 (42.5)	1127 (45.7)	1828 (40.7)

**Sex**			
Male	2526 (36.2)		
Female	4470 (63.8)		

**Schooling (years)**			
6 to 8	4496 (65.3)	1692 (69.7)	2684 (62.8)
9 to 11	2481 (34.7)	748 (30.3)	1666 (37.2)

**Hungry**			
Never	3428 (47.4)	1168 (47.0)	2138 (47.8)
Rarely	455 (5.9)	147 (5.5)	295 (6.2)
Sometimes	2548 (37.0)	877 (36.8)	1565 (36.9)
Most of the times or always	676 (9.7)	260 (10.7)	380 (9.0)

**Drank alcohol**			
Yes	984 (16.6)	410 (20.5)	536 (14.5)
No	5132 (83.4)	1638 (79.5)	3305 (85.5)

**Number of times bullied**			
0	3811 (60.4)	1203 (57.0)	2462 (62.3)
1 or 2	1381 (22.6)	457 (22.2)	872 (23.0)
3+	1017 (17.0)	424 (20.8)	546 (14.7)

**Most students kind and helpful**			
Never	1587 (25.2)	605 (28.7)	916 (23.2)
Rarely	705 (10.9)	284 (13.2)	395 (9.7)
Sometimes	2334 (36.7)	746 (34.8)	1507 (37.9)
Most of the times	852 (13.0)	254 (11.4)	563 (13.8)
Always	895 (14.2)	254 (11.8)	600 (15.3)

**Parents checked homework**			
Never	2091 (31.3)	744 (32.8)	1259 (30.4)
Rarely	515 (7.8)	219 (9.7)	278 (6.8)
Sometimes	1710 (26.7)	536 (24.6)	1003 (25.0)
Most of the times	922 (14.1)	249 (11.2)	414 (10.1)
Always	1763 (26.8)	476 (21.7)	1116 (27.6)

**Parents understood problems**			
Never	1583 (24.1)	565 (25.6)	952 (23.2)
Rarely	547 (8.3)	264 (11.8)	263 (6.4)
Sometimes	1710 (26.7)	600 (27.1)	1033 (26.4)
Most of the times	922 (14.1)	276 (12.8)	617 (15.0)
Always	1763 (26.8)	517 (22.8)	1175 (28.9)
Parental supervision			
Never	1807 (27.5)	643 (28.8)	1073 (26.4)
Rarely	510 (7.6)	218 (9.6)	279 (6.7)
Sometimes	1785 (27.3)	620 (27.3)	1106 (27.7)
Most of the times	925 (14.3)	291 (13.2)	595 (14.9)
Always	1539 (23.3)	467 (21.1)	1002 (24.3)

**Truancy**			
Yes	1405 (21.6)	605 (27.5)	723 (17.9)
No	5200 (78.4)	1638 (72.5)	3374 (82.1)

n* unweighted frequency

(%)** weighted percent

### Factors associated with truancy

As shown in Table 3, males were more likely to report truancy than females [OR = 1.22 (95% CI 1.16, 1.28)]. We also found the following factors as positively associated with history of truancy: being 14 years old, being in the 6^th ^to 8^th ^year of schooling, sometimes going hungry, drinking alcohol, perception that most students were rarely or sometimes kind and helpful, parents who rarely checked homework, parents who rarely understood problems and worries, and parents who rarely supervised their adolescents.

**Table 3:** Factors associated with truancy among adolescents in Swaziland

**Factor**	OR (95% CI)*
**Age**	
< 14	0.92 (0.85, 1.00)
14	1.11 (1.04, 1.19)
15+	1

**Sex**	
Male	1.22 (1.16, 1.28)
Female	1

**Schooling (years)**	
6 to 8	1.07 (1.01, 1.13)
9 to 11	1

**Hungry**	
Never	0.82 (0.75, 0.89)
Rarely	0.72 (0.62, 0.84)
Sometimes	1.30 (1.19, 1.41)
Most of the times or always	1

**Drank alcohol**	
Yes	1.34 (1.27, 1.42)
No	1

**Number of times bullied**	
0	0.74 (0.70, 0.79)
1 or 2	1.06 (0.98, 1.14)
3+	1

**Most students kind and helpful**	
Never	0.85 (0.77, 0.93)
Rarely	1.46 (1.30, 1.64)
Sometimes	1.11 (1.03, 1.21)
Most of the times	0.94 (0.84, 1.06)
Always	1

**Parents checked homework**	
Never	0.98 (0.89, 1.07)
Rarely	1.22 (1.06, 1.40)
Sometimes	1.05 (0.96, 1.15)
Most of the times	1.03 (0.91, 1.16)
Always	1

**Parents understood problems**	
Never	0.92 (0.83,1.01)
Rarely	1.50 (1.32, 1.71)
Sometimes	0.95 (0.87, 1.04)
Most of the times	0.87 (0.87, 0.98)
Always	1

Parental supervision	
Never	1.05 (0.96, 1.15)
Rarely	1.34 (1.17, 1.53)
Sometimes	0.98 (0.90, 1.07)
Most of the times	0.90 (0.80, 1.01)
Always	1

OR (95%CI)* adjusted for all the factors in the model

Adolescents who were 14 years old were more likely to report truancy than those of age 15 years or more [OR = 1.11 (95% CI 1.04, 1.19)]. Meanwhile, adolescents who were in their 6^th ^to 8^th ^year of schooling were 7% (OR = 1.07, 95%CI 1.01, 1.13) more likely to report truancy than those who were in their 9^th ^to 11^th ^year of schooling. The odds of truancy in adolescents who went hungry sometimes compared to those who were hungry most of the times were 1.30 (95% CI 1.19,1.41)times.

Compared to participants who did not drink alcohol, those who drank alcohol were more likely to report truancy [OR1.34 (95%CI 1.27, 1.42). Furthermore, adolescents who felt that most students were rarely or sometimes kind and helpful were likely to report truancy compared to those who felt that most students were always kind and helpful.

Adolescents who had parents who rarely checked their homework were more likely to report truancy than those whose parents always checked their homework [OR 1.22 (95%CI 1.06, 1.40)]. Adolescents who indicated that their parents rarely understood their problems and worries were 50% (OR = 1.50, 95%CI 1.32, 1.71) more likely to report truancy than those who said that their parents always understood their problems and worries. Finally, adolescents who were rarely supervised by their parents were more likely to report truancy than those who were always supervised by their parents [OR, 1.34 (95%CI 1.17, 1.53)].

### Protective factors for truancy

We identified the following protective factors for truancy: being well-fed, not being bullied, most students being kind and helpful to their schoolmates and parents most of the time understanding adolescents' problems and worries (Table 3).

Adolescents who never or rarely went hungry were 18% (OR = 0.82, 95%CI 0.75, 0.89) and 28% (OR = 0.72, 95%CI 0.62, 0.84), respectively, less likely to report truancy compared to those who most of the time or always went hungry. Compared with adolescents who were bullied at least three times, adolescents who were never bullied were 26% (OR = 0.74, 95%CI 0.70, 0.79) less likely to report truancy.

Lastly, adolescents who felt that their parents most of the time understood their problems and worries were 13% (OR = 0.87, 95%CI 0.78, 0.98) less likely to report truancy compared to those who felt that their parents never or only sometimes understood their problems and worries.

## Discussion

Our study, using a national sample of in-school adolescents in Swaziland, found that the prevalence of truancy was 21.6% (Table 2). We also found that self reported history of truancy was associated with lower school grade, having been victim of bullying, having gone hungry sometimes because of lack of food at home and consumption of alcohol. Adolescents who reported parental supervision most of the times or sometimes were less likely to have been truant compared to those who report no supervision.

The association between having gone hungry because of lack of food at home and being truant could be explained in several ways. First, it is possible that adolescents from poor households may miss class because they need an opportunity to fend for themselves. This could be done through begging or scrounging for food. Truant and hungry students may also be involved in piece work to earn some money to purchase food. Finally, the lack of food at home may just be a marker of many other social dysfunctions within the home.

In a national United States sample of adolescents 8^th ^and 10^th ^graders who are typically 13 to 16 years, Henry estimated a 4 week truancy prevalence of 10.5% to 16.4% in 2003 [7]. In comparison to US adolescents reported by Henry [7] our estimates were much higher. While we found that males were more likely to be truant than females, MacGillivary and Erickson, in a report on the school system in Denver, Colorado (United States) reported that there was no gender difference in truancy among adolescents [13]. Our findings that males were more likely to be truant could be a manifestation of cultural expectations. It is plausible that truancy among boys may be more tolerated than truancy among girls/girls being truant.

We found that adolescents who reported parental supervision and support were less likely to be truant than those who lacked these social supports. A similar finding was reported by Stanton et al [14] who reported that parental support towards adolescents was associated with a protective effect against unhealthy and antisocial behaviours.

Adolescents who reported never having been bullied were less likely to have been truant. 1979 study by Nielsen and Gerber [15] reported that truancy was associated with fear of peers. In a situation where the adolescent is victim of bullying, fear of other students may facilitate truant behavior i.e. the adolescent is running away from bullies. This calls for school administrators and teachers to be vigilant against situations that promote or facilitate bullying behaviours among students. This can be achieved through setting-specific measures tailored to the age and grades of pupils and the socio-cultural environment of a particular setting.

We also found that lower school grades but not age were associated with a history of truancy. This could suggest possible laxity of behavior amongst lower grades students probably as a result of lower school expectations from their teachers or themselves. Grades that are within secondary school system were associated with less likelihood of being truant.

Many other studies on adolescents have reported the association between adolescents truant behaviour and alcohol use [3,5,16]. Alcohol or truancy may be just a marker of other socially dysfunctional behaviours. It is also possible that the unsupervised free time that truant adolescents have may make them more likely to experiment with alcohol than if they were in school.

Vreeman and Caroll [17] have reported a systematic review of the literature in which they assessed the effectiveness of different school-based interventions against bullying. These authors found that interventions which included increasing social workers in school and promoted mentoring of students were successful in reducing the prevalence of bullying. Also these authors found that interventions were effective in reducing bullying in some settings but not much so in others, possibly suggesting site-specific effects.

Some of these measures may be putting in place interventions that promote family-friendly schools. The components of family friendly schools may include establishment of parent liaison officer, regular parent-teacher contact, ensuring that parents assist in homework and encouraging parental decisions in school administration [18]. With family friendly schools, parents should inform teachers of reasons for adolescents missing school and teachers should inform parents of any absences. There is also need to encourage parental supervision of adolescents.

While truancy could be a consequence of poor academic performance, it is also possible that it can result into poor school performance. This could have long term effects where, because of lack of education, the adolescents' future and especially jobs prospects are uncertain. El-Ibiary and Youmans have reported that women in the United States needed to have high school level reading ability to understand consumer advice on contraceptive packs [19]. Foster et al [20] have also reported that in California, women without high school level education had difficulties in understanding information about emergency contraception compared to women with high school education.

### Limitations of the study

Our study had a number of limitations. Data were collected via a self-completed questionnaire. As information on truancy may be potentially sensitive, it is possible that some of the study participants may have under-reported intentionally. Although questionnaires were completed anonymously, this was done in class under supervision of a research assistant. It is also possible that some study participants may have misreported because of recall problem. The question on truancy specifically asked if the study participants had missed school without permission within the last 30 days. Adolescents who had missed school within that period but thought they had missed school longer than the stated period, or adolescents who missed school longer than 30 days prior to survey but thought they had missed school recently, all had potential to misreport.

While it is possible to collect useful information on truancy based on self-reported information, the quality of information can be strengthened by use of official school attendance data. The Global School Health Survey does not collect such data. Predictor variables such as age, academic school performance, parental characteristics could also be obtained through official records which may be much more reliable than self reported data. However such an exercise may require that data are already available for administrative purpose.

Because our study was based on secondary analysis of existing data, we had no control on other potentially useful variables that may have been assessed but were not collected in the Swaziland Global School-Based Health Survey. Truancy has also been reported to be associated with violence at or near school, association with truant friends, lack of family support for regular attendance, weapon carrying, emotional or mental health problems, lack of a clear path to more education or work and inability to keep pace with academic requirements [5,21,22]. These variables were not available within the Swaziland GSHS.

## Conclusion

We are unaware of any previous studies that have reported on prevalence of truancy and its predictors in Swaziland. Published reports on truant behaviours in southern Africa are limited. In this regard therefore, our study could be the first to have reported on this psychosocially problematic behaviour in Swaziland. With a prevalence of about 21% among girls and boys, truancy should be a major social concern in Swaziland. We suggest that efforts aimed to reduce truant behaviours should incorporate our understanding of the factors that are associated with the behaviour.

## Abbreviations

CDC: Centers for Disease Control and Prevention

GSHS: Global School-Based Health Survey

HIV: human immunodeficiency virus

UNAIDS: Joint United Nations Program on HIV/AIDS

UNICEF: United Nations Children's Fund

UNESCO: United Nations Educational, Scientific and Cultural Organisation

WHO: World Health Organisation

## Competing interests

The author(s) declare that they have no competing interests.

## Authors' contributions

SS analysed data and participated in drafting of manuscript.

ASM conceived the analysis plan, participated in the interpretation and drafting of manuscript.

ER participated in the interpretation and drafting of manuscript.

All the authors agreed to the final draft of the manuscript.
